# Sustaining Edible Grass (*Rumex patientia* L. × *Rumex tianschanicus* Losinsk.) Through Summer Lethal Stress: Multi-Omics Reveals Shading-Mediated Mitigation of High Light-Aggravated Heat Damage

**DOI:** 10.3390/antiox15010033

**Published:** 2025-12-25

**Authors:** Zengyang He, Qinzhuo Zhong, Xinyao Li, Miaofen Chen, Wei Liu, Tao Jiang, Jianfeng Zou

**Affiliations:** 1 Hunan Key Laboratory of Traditional Chinese Veterinary Medicine, Hunan Agricultural University, Changsha 410128, China; 152550hzy@stu.hunau.edu.cn (Z.H.); Miaofen_Chen@hunau.edu.cn (M.C.); weiliu@hunau.edu.cn (W.L.); 2 College of Horticulture, Hunan Agricultural University, Changsha 410128, China; zhongqinzhuo@stu.hunau.edu.cn (Q.Z.); xinyaolin@stu.hunau.edu.cn (X.L.); 3 College of Veterinary, Hunan Agricultural University, Changsha 410128, China; 4 Academician Workstation, Jiangxi University of Chinese Medicine, Nanchang 330004, China

**Keywords:** edible grass, shading treatment, high light-aggravated heat stress, transcriptome, metabolome

## Abstract

Edible Grass (EG) is a hybrid vegetable variety valued for its high biomass and protein content, garnering significant interest in recent years for its potential in food, feed, and health product applications. However, in subtropical climates, intense light and high temperatures severely affect the growth and development of Edible Grass (EG), leading to substantial reductions in yield and quality. This study was conducted in the subtropical humid monsoon climate zone of Changsha, Hunan, China, comparing two growth conditions: natural light (CK) and shading treatment (ST). High light-aggravated heat damage under CK significantly reduced EG yield and quality (*p* < 0.05), with severe cases leading to plant death. and could even lead to plant death in severe cases. Specifically, maximum air and leaf temperatures under CK reached 38.85 °C and 38.14 °C, respectively, well exceeding the plant’s optimal growth range. Shading treatment (ST) effectively alleviated this damage, significantly increasing the net photosynthetic rate, stomatal conductance, and intercellular CO_2_ concentration, while decreasing leaf temperature and transpiration rate (*p* < 0.001). The analysis of physiological and biochemical indicators indicates that after ST, the activities of SOD, CAT, and POD in the leaves decreased, while the contents of MDA and H_2_O_2_ were significantly lower compared to the CK group (*p* < 0.001). The transcriptome sequencing results indicate that a total of 8004 DEGs were identified under shading treatment (ST) relative to natural light (CK), with 3197 genes upregulated and 4807 genes downregulated. Significantly enriched Gene Ontology (GO) terms include ‘cell membrane’, ‘extracellular region’, and ‘protein kinase activity’, while significantly enriched KEGG metabolic pathways include ‘plant hormone signal transduction’, ‘photosynthesis–antenna proteins’, and ‘glutathione metabolism’. Compared to CK, the expression of genes associated with oxidative stress (e.g., *CAT1*, *OXR1*, *APX*, *GPX*) was significantly downregulated in ST, indicating a relief from light-aggravated heat stress. This transcriptional reprogramming was corroborated by metabolomic data, which showed reduced accumulation of key flavonoid compounds, aligning with the downregulation of their biosynthetic genes as well as genes encoding heat shock proteins (e.g., Hsp40, Hsp70, Hsp90). It indicated that plants switch from a ‘ROS stress–high energy defense’ mode to a ‘low oxidative pressure–resource-saving’ mode. Collectively, ST significantly alleviated the physiological damage of forage grasses under heat stress by modulating the processing of endoplasmic reticulum heat stress proteins, plant hormones, and related genes and metabolic pathways, thereby improving photosynthetic efficiency and yield. The findings provide a theoretical basis for optimizing the cultivation management of EG, particularly in subtropical regions, where shade treatment serves as an effective agronomic strategy to significantly enhance the stress resistance and yield of EG.

## 1. Introduction

**Figure 1:**
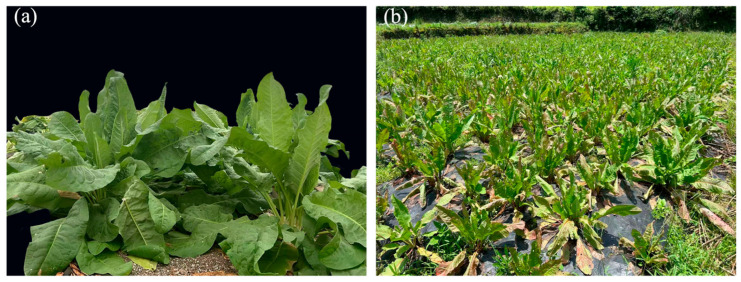
Growth Morphology of edible grass Under Natural Light Conditions (Control, CK) and Shading Treatment (ST). (**a**): Leaf morphology of edible grass in spring, photographed on 15 April 2022. (**b**): Leaf morphology of edible grass in summer, photographed on 1 July 2022.

Edible Grass (EG), *Rumex patientia* L. × *Rumex tianschanicus* Losinsk., is a hybrid vegetable variety that has been approved as a novel food resource [1]. Valued for its high biomass and protein content, this crop has gained increasing attention for potential use in food, feed, and health products in recent years [2,3,4]. However, research on EG remains limited, particularly regarding its cultivation, stress responses, and pest control of EG, with only Ślesak et al. [5] reporting on micropropagation protocols for similar plants, and He et al. [6] recently reporting the optimal nitrogen supply levels during the seedling stage of edible grass under hydroponic conditions. In field cultivation, the perennial nature and the ability to be harvested repeatedly throughout the year have become key selling points for seedling suppliers. Nevertheless, the growth and development of this plant is significantly restricted by the strong light and high temperatures of subtropical regions during the summer (Figure 1b). Based on this phenomenon, we hypothesize that high light-aggravated heat stress greatly limits the growth and development of EG.

The growth and yield of plants can be adversely affected by abiotic stress, and the effects of these stresses are becoming increasingly pronounced due to climate change, either directly or indirectly. Plants exhibit a range of responses to abiotic stress, which include changes in gene expression, alterations in physiological functions, modifications in plant structure, as well as adjustments in both primary and secondary metabolic processes. These intricate alterations enable plants the capacity to endure and/or adjust to such challenges. The duration and intensity of stress, the plant’s genotype, the interplay of various stressors, the specific tissues and cells involved, and the growth stages at which the plant perceives the stress may all influence the intricacy of its responses [7].

Plants are adversely affected by high temperatures and have evolved complex mechanisms to cope with heat stress [8]. Heat stress impacts photosynthesis [9], respiration [10], and water metabolism [11], leading to reduced photosynthetic efficiency and productivity. Furthermore, elevated temperatures can damage the structures of chloroplasts and photosynthetic systems, resulting in the accumulation of reactive oxygen species (ROS). Heat stress triggers oxidative stress and lipid peroxidation in plants. To enhance their heat tolerance, plants increase the activity of antioxidant enzymes such as superoxide dismutase (SOD), ascorbate peroxidase (APX), catalase (CAT), glutathione reductase (GR), and peroxidase (POX). Furthermore, heat stress can lead to dehydration, adversely affecting plant growth and development [12]. By modifying agronomic practices, it is anticipated that the impacts of high temperatures can be mitigated [13]. Artificial shading is a strategy that mitigates the combined stress of intense light and high temperature by reducing the radiative heat load on the plant canopy. This approach includes altering sowing timings, increasing nutrient supply utilizing various plant growth regulators, and employing techniques such as nano-biochar substitutes and microbial inoculation. Collectively, these methods promote plant growth in environments with extreme high temperature [14]. Typically, shade cloth or shade nets are used to alleviate heat stress. Shade nets can be black, green, white, or reflective aluminum, with a shading rate typically ranging from 20% to 30% [14]. Shading is generally implemented during the hottest periods or when the plants are most sensitive to heat, such as during the fruit development phase of economic crops [15]. Therefore, promoting and applying protective shading in regions like the subtropics and tropics is a crucial agronomic measure to alleviate high light-aggravated heat stress and enhance the yield and quality of EG.

In the context of heightened light-aggravated heat stress under natural light conditions, this study compares the morphological characteristics, yield, quality, antioxidant properties, and gene expression of EG. Under open control conditions (CK) and shading treatment (ST). The aim is to clarify the impact of shading on the yield and quality of EG. Specifically, we seek to elucidate the physiological, biochemical, and molecular mechanisms by which shading, by modulating the light and thermal environment, alleviates light radiation-induced heat stress and enhances EG productivity. Heat stress under natural light conditions is a prevalent phenomenon in field environments, while shading treatment serves as an effective mitigation measure to reduce light intensity and ambient temperature.

## 2. Materials and Methods

### 2.1. Plants Material

The seeds of EG were supplied by the Beijing Junxinquansheng Environmental Science and Technology Research Institute (Beijing, China). The cultivation experiment was conducted at the Hunan Agricultural University planting base, located at geographical coordinates 28.18° N, 113.11° E, with an altitude of 44.9 m. The row spacing for the EG was set at 50 cm, with a plant spacing of 32 cm, resulting in a density of 6.25 plants per square meter. Throughout the entire growth period, only manual weeding and standard irrigation practices were employed. A completely randomized block design was utilized, comprising two treatments: natural light (Control, CK) and shading treatment (ST). The experiment included a total of 10 plots, with each plot serving as an independent biological replicate. The shading treatment was implemented from 20 June to 30 June 2022. For the ST group, a single layer of black shading net was installed at a height of 1.5 m above the ground, covering the designated plots, with a shading rate of the net was 30%. All other environmental conditions were kept identical between the CK and ST groups, with the plants in the CK group remained under open-air conditions without any shading.

### 2.2. Growth Indicators and Yield Measurement

Given that Edible Grass is a perennial crop grown for multiple harvests (similar to *Allium tuberosum*), a uniform cutting was performed prior to the application of shading treatments. This practice is a standard management method for this crop, aimed at synchronizing plant regrowth and ensuring that the measured effects are attributable to the experimental treatments rather than pre-existing differences in plant size or developmental stage. Plots were then selected based on the fresh weight after this standardized cutting to ensure consistent initial biomass for the experiment. These were six plants in each plot to ensure similar fresh grass weights (382.45 ± 34.26 g/stem). EG samples were collected from the different treatments to determine plant height, leaf width, number of leaves, and the fresh weight of the aerial parts. Six plants were selected from each plot for measurement. The fresh weight yield per hectare (tons/hectare) was then calculated by multiplying the average fresh weight of each plant by the plant density (62,500 plants/hectare, equivalent to a planting density of 6.25 plants/m^2^). For transcriptome analysis, leaf samples were rapidly frozen in liquid nitrogen and stored at −80 °C.

The dry matter (DM) and crude protein (CP) contents of the dried EG powder were analyzed in accordance with the standard methods specified by the Association of Official Analytical Chemists (AOAC) [16]. The method proposed by Van Soest et al. [17] was adopted for the determination of neutral detergent fiber (NDF) and acid detergent fiber (ADF). With amylase and anhydrous sodium sulfite being used in this study.

### 2.3. Determination of Photosynthetic Parameters

The net photosynthetic rate, stomatal conductance, intercellular CO_2_ concentration, transpiration rate, air temperature, and leaf temperature of EG were measured using a LI-6400XT portable photosynthesis system (LI-COR, Lincoln, NE, USA). Measurements were conducted on sunny days from 14:30 to 15:30, simultaneously for both natural light (CK) and shading treatment (ST) groups. The measurements were taken on mature leaves located in the middle part of the plant. Six biological replicates (individual plants) were measured for each treatment to obtain average values for analysis. The determination of photosynthetic parameters for EG was based on the method of He et al. [6], and the third fully expanded functional leaf from the top was selected for measurement.

### 2.4. Biochemical and Physiological Analysis

Biochemical and physiological analyses were conducted on the EG leaves collected from the CK and ST groups. The activities of superoxide dismutase (SOD), ascorbate peroxidase (APX), peroxidase (POD), and catalase (CAT) were measured. Quantitative analyses were performed for the contents of malondialdehyde (MDA) and hydrogen peroxide (H_2_O_2_). The quantification of compound substances and enzyme activities were assessed using kits provided by the Nanjing Jiancheng Bioengineering Institute in China, in accordance with the manufacturers’ instructions. The ·O_2_^−^ scavenging property was tested using a Superoxide Anion Activity Content Assay Kit (Solarbio, Shanghai, China), following the manufacturer’s instructions.

### 2.5. RNA Extraction and Transcriptome Sequencing

Total RNA was extracted from the leaf samples of both the control (CK) and shading treatment (ST) groups using a commercial RNA extraction kit (Meiji Bio-pharmaceutical Technology, Shanghai, China), following the manufacturer’s instructions. The concentration and purity of the extracted RNA were assessed using a NanoDrop ND-2000 spectrophotometer (NanoDrop Technologies, Wilmington, DE, USA). RNA integrity was confirmed by 1.5% agarose gel electrophoresis.

Sequencing libraries were prepared using the Illumina TruSeq Stranded mRNA LT Sample Prep Kit according to the standard protocol. The qualified libraries were sequenced on an Illumina NovaSeq 6000 platform by MajorBio Bio-Pharm Technology Co., Ltd. (Shanghai, China), generating 150 bp paired-end reads.

Since no reference genome is available for EG, a de novo transcriptome assembly was performed to identify genes and transcripts. Briefly, high-quality clean reads from all samples were pooled and assembled into transcripts using the Trinity software (version 2.15.1) with default parameters. To reduce redundancy arising from allelic variants (due to the hybrid nature of EG) and alternative splicing, the assembled transcripts were further clustered using the CD-HIT-EST tool (version 4.8.1) with a sequence identity threshold of 95%. This step aimed to merge transcripts likely originating from the same gene locus. Subsequently, the clustered transcripts were processed with TGICL to obtain a final set of non-redundant unigenes. The assembled transcripts were then clustered to obtain non-redundant unigenes using TGICL. For gene identification and functional annotation, these unigenes were aligned against several public databases, including the NCBI non-redundant (NR, version 2023.07) protein database, Swiss-Prot (version 2023.11), Gene Ontology (GO, version 2023.07), Kyot Encyclopedia of Genes and Genomes (KEGG, version 2023.09), and eggNOG (http://eggnog5.embl.de/, accessed on 10 July 2025) using BLASTX (version 2.9.0) with a significance threshold of E-value < 1 × 10^−5^. Based on the homology information of the best hit in the NR database (primarily from well-annotated model plants such as *Arabidopsis thaliana*, *Oryza sativa*, and *Solanum lycopersicum*), functional annotations including Gene Ontology (GO) terms and Kyoto Encyclopedia of Genes and Genomes (KEGG) pathways were assigned to each unigene through an annotation transfer process.

### 2.6. Extraction and Determination of Metabolites

EG leaf tissue samples (100 mg) were selected, ground with liquid nitrogen, and subsequently suspended in pre-cooled 80% methanol solution. After thorough mixing on a plate vortex mixer, the samples were statically incubated at 4 °C in an ice bath for 5 min. Following incubation, centrifugation was performed at 15,000× *g* for 20 min at 4 °C. A portion of the supernatant was collected and diluted with chromatography-grade water to adjust the final methanol concentration of the system to 53%. The diluted samples were transferred to new centrifuge tubes and centrifuged again under the same parameters (4 °C, 15,000× *g*) for 20 min. Finally, the supernatant was collected for liquid chromatography-tandem mass spectrometry (LC-MS/MS) detection [18].

### 2.7. Data Analysis

The data on growth physiology and antioxidant characteristic indicators were organized using Microsoft Excel 2016 and analyzed through the independent samples *t*-test procedure in IBM SPSS Statistic (Version 25.0.0).

An analysis of transcriptome data using bioinformatics was performed on the Majorbio Cloud Platform (www.MajorBio.com (accessed on accessed on 10 July 2025)). Data analysis was performed using DESeq2 (Version 1.42.0) (https://bioconductor.org/packages/DESeq2/, accessed on 10 July 2025) [19]. DEGs were defined as |log_2_(fold change)| ≥ 1 and false discovery rate (FDR) significance score (adjusted *p*-value) < 0.05. The biological functions and associated pathways of all DEGs were identified through annotations from the Gene Ontology (GO) and Kyoto Encyclopedia of Genes and Genomes (KEGG) databases. GO enrichment analysis of these genes was carried out using Gotools software (https://github.com/tanghaibao/GOatools, accessed on 10 July 2025) [20]. KEGG pathway enrichment analysis was performed using R (version 4.3.0) (accessed on 10 July 2025) and relevant R packages.

Metabolomic data analysis was performed on the Majorbio Cloud Platform (www.MajorBio.com, accessed on 15 October 2025). To clarify the differences in metabolite composition among different samples, Principal Component Analysis (PCA) was performed on all metabolites. The screening criteria for differential metabolites (DAMs) were set as a *p*-value < 0.05 and |Fold Change| > 2.

Prism 9.0 software was used to generate heat maps of DEGs using z-score normalization.

## 3. Results

### 3.1. The Impact of Shading Treatment on the Yield and Quality of Edible Grass

Table 1 shows the effects of shading treatment on the yield and quality of edible grass. In the ST group compared to the CK group, plant height, leaf weight, fresh weight, dry weight, nitrogen content, and crude protein content were significantly increased. (*p* < 0.001). Conversely, the contents of dry matter, NDF, and ADF were significantly reduced under ST relative to CK (*p* < 0.001).

### 3.2. The Impact of Shading Treatment on the Antioxidative Indices of Edible Grass

Table 2 shows the antioxidant indicators in the control (CK) and shading treatment (ST) groups. The results indicate that the activities of the antioxidant enzymes SOD, APX, POD, and CAT were significantly higher in the CK group than in the ST group (*p* < 0.001). Conversely, the levels of oxidative stress markers, including MDA, H_2_O_2_, and ·O_2_^−^ were also significantly elevated in the CK group compared to the ST group (*p* < 0.001).

### 3.3. The Impact of Shading Treatment on the Photosynthetic Parameters of Edible Grass

Table 3 presents the photosynthetic parameters of edible grass under control (CK) and shading treatment (ST) conditions. Compared to the CK group, the ST group exhibited a significantly higher net photosynthetic rate, stomatal conductance, and intercellular CO_2_ concentration (*p* < 0.001). In contrast, the transpiration rate and leaf temperature were significantly lower in the ST group than in the CK group (*p* < 0.001). Air temperature at the time of measurement did not differ significantly between the two groups.

### 3.4. RNA-Seq Sequencing, Annotation, and Analysis

#### 3.4.1. RNA Isolation, Transcriptome Sequencing, and De Novo Assembly

The extracted RNA concentrations ranged from 210 to 409 ng/µL, with an optical density ratio of 2.06–2.12 at 260/280, indicating the high quality of the RNA samples. Illumina sequencing generated around 39 million reads for each sample, and following careful quality assessment, 99.3% of the data was preserved across the various sample groups linked to shading treatment (ST) and natural light (CK) (Table S1).

After strict filtering, high-quality sequence data from every sample were utilized for de novo transcriptome assembly. The initial assembly contained 279,651 transcripts, with an N50 value of 1979 bp (Table 4). The filtered assembly of transcripts removed low-quality transcripts with TPM (transcripts per million) values less than 1 and transcripts with 99% identity. A total of 192,766 transcripts were obtained from the filtered assembly, with lengths ranging from 201 to 21,819 bp (mean: 1130 bp). The N50 value of the filtered assembly was found to be 1769 bp (Table 4). It is important to note that, following the de novo assembly, we implemented a stringent clustering step (95% identity) to collapse allelic variants and highly similar sequences from the hybrid parents. Therefore, the final set of 86,785 unigenes (Table 4) more likely represents distinct gene loci, although we acknowledge that some closely related gene family members may remain unresolved.

#### 3.4.2. Quality Assessment and Annotation of the Filtering Component

The quality of the de novo assemblies was assessed by aligning the clean reads back to the assembled transcriptome itself. Approximately 85% of the filtered reads from each sample successfully mapped to the assembly (Table S2), indicating its high completeness and integrity.

Following assembly, the unigenes were functionally annotated by performing BLASTX searches against six public databases: Gene Ontology (GO), Kyoto Encyclopedia of Genes and Genomes (KEGG), evolutionary genealogy of genes: Non-supervised Orthologous Groups (eggNOG), NCBI non-redundant protein sequences (NR), Swiss-Prot, and Protein family (Pfam). This process resulted in 30,949, 16,342, 31,974, 38,541, 28,343, and 24,527 unigenes being annotated in the respective databases (Figure S1). Among these, the NR database provided annotations for the largest number of unigenes (38,541). Figure S3a illustrates the E-value distribution of the NR annotations, showing that 61.21% of the aligned unigenes had significant matches (E-value < 1 × 10^−30^). The similarity distribution (Figure S3b) indicates that 86.55% of the annotated unigenes shared greater than 60% sequence similarity with known proteins. Furthermore, the species distribution of the top BLAST hits (Figure S3c) revealed that the highest homology was with *Nepenthes gracilis* (13.75%), followed by *Beta vulgaris* (5.26%), *Chenopodium quinoa* (4.22%), *Carnegiea gigantea* (3.78%), and *Spinacia oleracea* (4.22%). Since EG lacks a closely related reference genome, the BLASTX alignment showed fragmentary sequence similarities with various dicot plants, with the highest proportion (13.75%) matching *Nepenthes gracilis*. This low level of homology confirms the necessity of performing de novo transcriptome assembly for this non-model species.

The annotation results of eggNOG are shown in Figure S2**,** where 31,974 unigenes are distributed across 24 major categories. The primary functional categories, excluding the category of Function Unknown, include posttranslational modification, protein turnover, chaperones, signal transduction mechanisms, and replication, recombination, and repair.

Gene Ontology (GO) annotation successfully assigned functional terms to 70,008 sequences were successfully annotated to 5370 GO terms. Among these, the ‘Cellular Component’ category under the ‘Cell’ class is the most representative, followed by ‘Biological Process’, ‘Single Biological Process’, ‘Molecular Function’, and ‘Catalytic Activity’ (Figure S2). A total of 30,949 unigenes were assigned to Gene Ontology (GO) annotations and categorized into three main categories: cellular components (53,817 terms, accounting for 17.08%), molecular functions (92,168 terms, accounting for 29.24%), and biological processes (169,152 terms, accounting for 53.68%) (Figure S2). The top three biological processes are macromolecule metabolic process, cellular nitrogen compound metabolic process, and organic cyclic compound metabolic process.

#### 3.4.3. Quantification and DEGs and Transcripts

**Figure 2:**
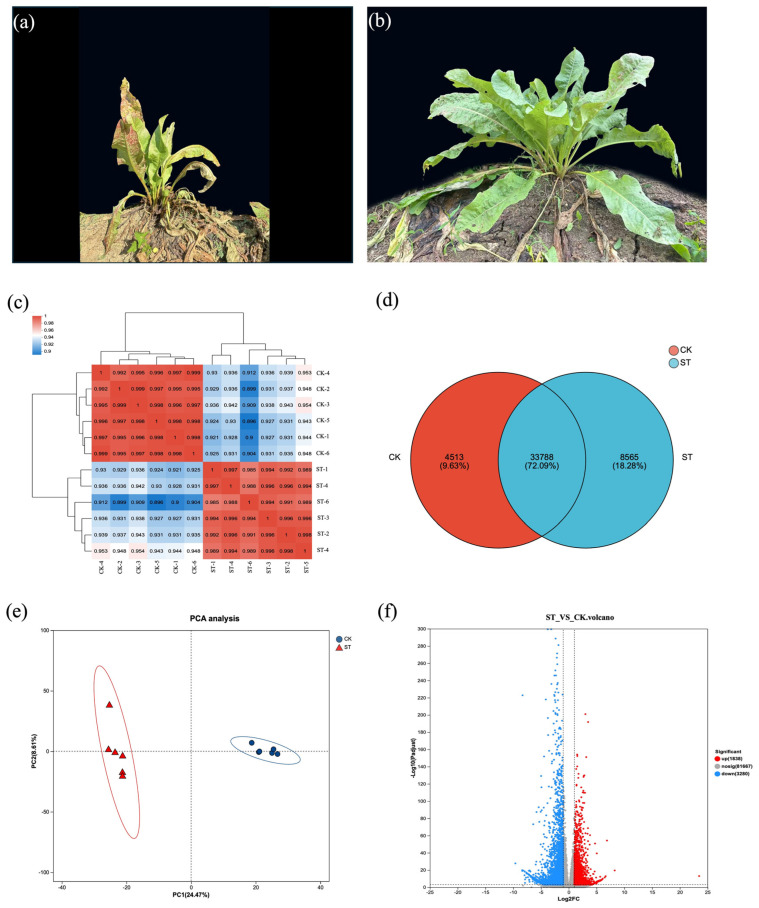
Overview of transcriptome analysis for edible grass under control and shading treatment. (**a**) Representative plants from the control group (CK) under natural light, photographed on 25 July 2022. (**b**) Representative plants from the shading treatment group (ST), photographed on 25 July 2022. (**c**) Heatmap depicting the correlation coefficients between all samples from the CK and ST groups, demonstrating the reproducibility within groups and distinction between treatments. (**d**) Venn diagram showing the number of unique and shared unigenes expressed in the CK and ST groups. (**e**) Principal Component Analysis (PCA) plot of all samples based on gene expression profiles, highlighting the clear separation between the CK and ST groups. (**f**) Volcano plot visualizing the DEGs. The plot displays the statistical significance (−log10 adjusted *p*-value) versus the magnitude of change (log2 fold change) for each gene. Significantly upregulated (red) and downregulated (blue) genes are highlighted, with non-significant genes shown in gray.

Transcripts exhibiting a TPM value of at least 1 are regarded as expressed. Under natural light, the leaves of the herbaceous plant contain 4513 unique unigenes, whereas under shading treatment, there are 8565 unique unigenes, with a total of 33,788 unigenes shared between the two groups (Figure 2d). Figure 2c,e indicates a significant separation between the samples of the ST group and the CK samples, providing support for subsequent gene analysis.

To identify DEGs that exhibiting significant changes under natural light and shading treatments, we analyzed the libraries from the ST group and the CK group. A total of 8004 filtered single genes (*p*-adjust ≤ 0.05 and |log2FC| ≥ 1) showed differential transcriptional evidence of varying lengths. In the comparison of ST vs. CK, more genes were downregulated than upregulated. A total of 3197 genes were upregulated in ST relative to CK, and 4807 genes were downregulated in ST relative to CK (Figure 2f).

**Figure 3:**
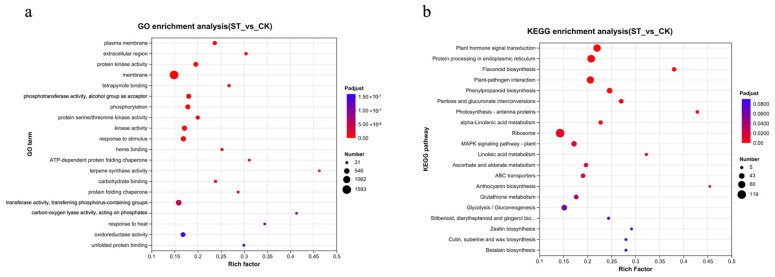
Enrichment analysis of DEGs in response to shading treatment. (**a**) Gene Ontology (GO) enrichment analysis bubble chart. The chart displays the most significantly enriched GO terms across the three ontologies: Biological Process (BP), Cellular Component (CC), and Molecular Function (MF). The size of the bubble represents the number of DEGs in that term, and the color indicates the level of statistical significance (−log10(adjusted *p*-value)). (**b**) Kyoto Encyclopedia of Genes and Genomes (KEGG) pathway enrichment analysis bubble chart. The chart shows the most significantly enriched metabolic and signal transduction pathways. Bubble size corresponds to the number of DEGs mapped to the pathway, and color represents the enrichment significance (−log10(adjusted *p*-value)).

Further GO and KEGG enrichment analyses were conducted on the DEGs (Figure 3). A total of 3918 DEGs were annotated with GO terms. The top five significantly ranked GO terms were plasma membrane, extracellular region, protein kinase activity, membrane, and tetrapyrrole binding. Among these, the term ‘membrane’ had the highest number of enriched differential genes, totaling 1593 (accounting for 40.6%) (Figure 3a).

The results of the enrichment analysis of KEGG metabolic pathways (Figure 3b) are as follows: Plant hormone signal transduction, Protein processing in endoplasmic reticulum, Flavonoid biosynthesis, Plant-pathogen interaction, Photosynthesis–antenna proteins, MAPK signaling pathway–plant, ABC transporters, Glutathione metabolism, Glycolysis/Gluconeogenesis, and Cutin, suberine and wax biosynthesis are among the significantly enriched metabolic pathways.

**Figure 4:**
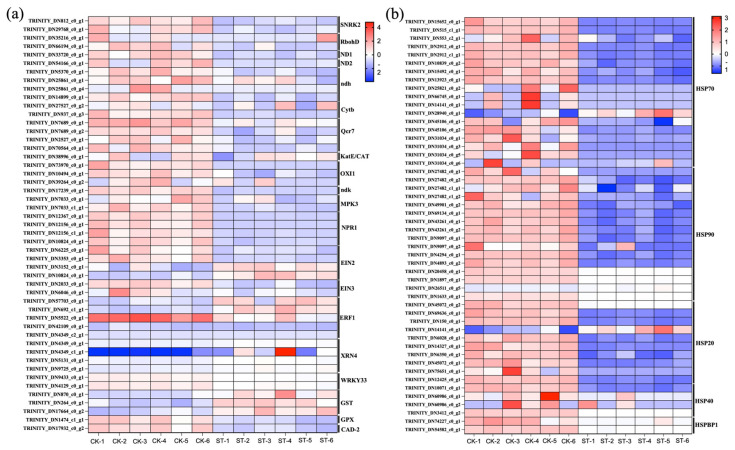
Expression patterns of key DEGs under shading treatment. (**a**) Heatmap showing the expression levels (Z-score normalized) of selected DEGs significantly enriched in the ‘Plant hormone signal transduction’, ‘Photosynthesis–antenna proteins’, and ‘MAPK signaling pathway–plant’ KEGG pathways. Key regulators are labeled, including ERF1, SNRK2, EIN3. (**b**) Heatmap showing the expression levels of DEGs related to heat stress response, enriched in the ‘Protein processing in endoplasmic reticulum’ KEGG pathway. Key heat shock protein (HSP) genes are labeled, including Hsp20, Hsp70, Hsp90. Each row represents a gene, and each column represents a biological replicate. Red color indicates higher expression relative to the mean, and blue color indicates lower expression.

Figure 4a illustrates the DEGs that are significantly upregulated or downregulated in the pathways of Plant hormone signal transduction, Photosynthesis–antenna proteins, and MAPK signaling pathway–plant. Figure 4b shows the DEGs that are significantly upregulated or downregulated in the pathway of Protein processing in endoplasmic reticulum.

### 3.5. Metabolite Analysis of Edible Grass Leaves

**Figure 5:**
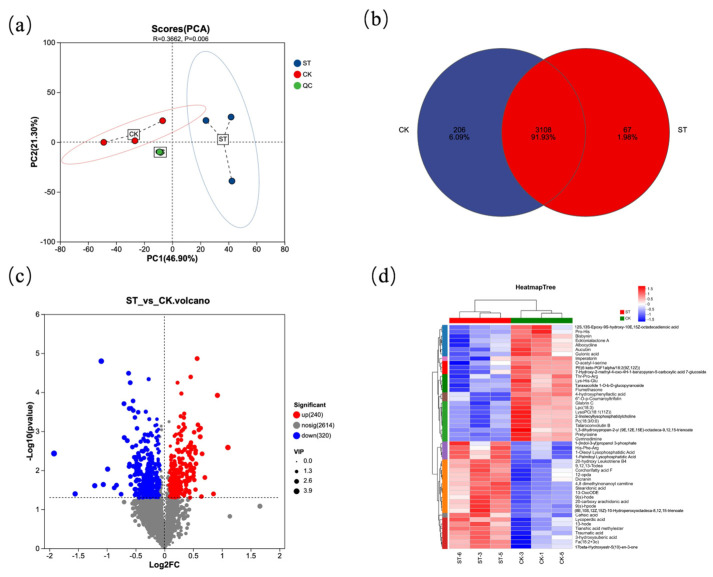
Statistical plot of differential metabolites. (**a**) Principal component analysis of the samples; (**b**) Venn diagram of differential metabolites from two groups; (**c**) Volcano plot visualizing of differential metabolites from two groups; (**d**) heatmap of the relative differential metabolites from two groups.

Figure 5 presents the analysis diagrams of differential metabolites between the natural light group (CK) and the shading treatment group (ST). Figure 5a shows the PCA plot among different samples, which intuitively reveals a significant separation between the CK and ST groups (R = 0.3662, *p* = 0.006). The Venn diagram (Figure 5b) indicates that there are 3108 common metabolites between the two groups, with 206 unique metabolites in the CK group and 67 unique metabolites in the ST group. In the volcano plot (Figure 5c), red dots represent 240 metabolites upregulated in CK relative to ST, while blue dots represent 320 metabolites downregulated in CK relative to ST. Figure 5d clearly reflects that the metabolites between the two groups can be divided into 10 subclusters.

Compared with the ST group, metabolites such as Pro-His, Bisbynin, and Ecklonialactone A were significantly higher in CK than in ST; in contrast, metabolites including 1-(Indol-3-yl)propanol 3-phosphate, 1-Oleoyl Lysophosphatidic Acid, and 1-Palmitoyl Lysophosphatidic Acid were significantly lower in CK than in ST.

**Figure 6:**
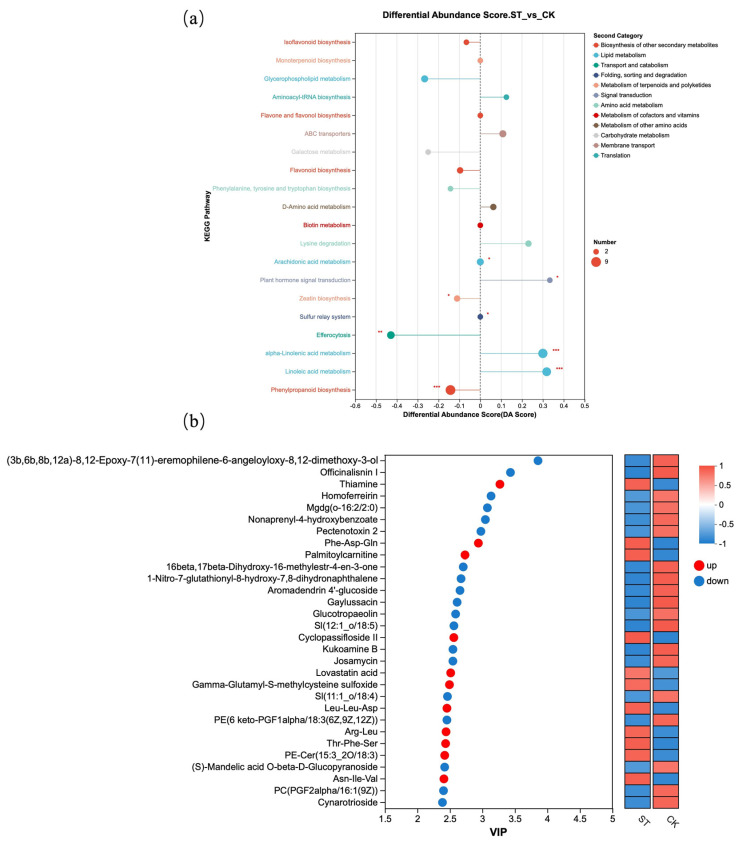
Metabolite KEGG enrichment analysis. (**a**) Commonly enriched metabolic pathways among the comparison groups; (**b**) VIP plot visualizing of differential metabolites from two groups.

Figure 6 presents further analysis based on differential metabolites. Figure 6a shows the DA Score of different metabolites in KEGG pathways. Compared with the natural light group (CK), the metabolite contents in KEGG metabolic pathways such as Isoflavonoid biosynthesis, Flavonoid biosynthesis, and Phenylpropanoid biosynthesis were lower; all three pathways belong to “Biosynthesis of other secondary metabolites” in the Second Category. Additionally, the Galactose metabolism pathway under Carbohydrate metabolism also exhibited lower metabolite levels. In contrast, compared with the CK group, the ST group showed higher metabolite contents in KEGG pathways including alpha-Linolenic acid metabolism and Linoleic acid metabolism, which are classified under Lipid metabolism. Figure 6b displayed the top 30 differential metabolites with the highest VIP scores between the CK and ST groups.

### 3.6. Combined Transcriptomic and Metabolomic Analysis of Edible Grass Leaves

**Figure 7:**
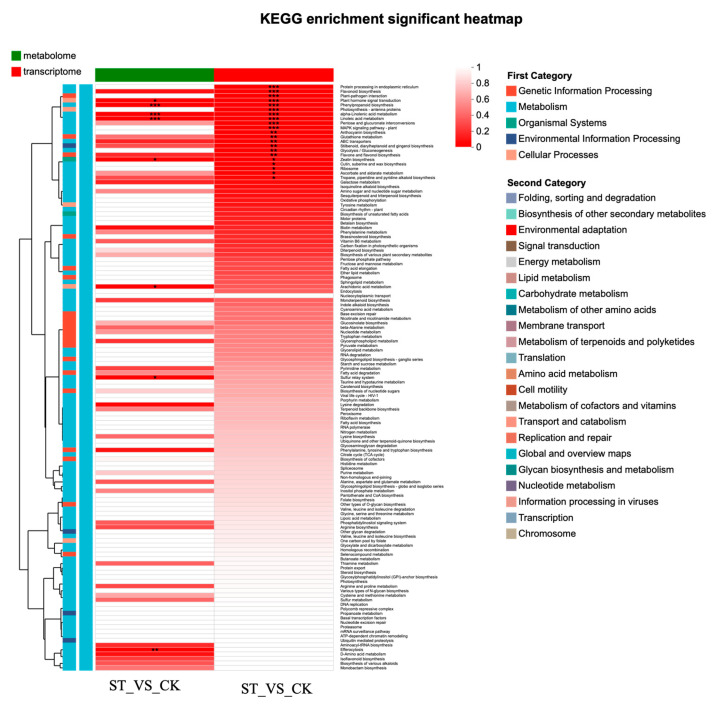
KEGG enrichment analysis heatmap based on combined transcriptomic and metabolomic analysis. Asterisks indicate statistical significance levels: * indicates a 0.01 < ** *p* < 0.05. *** indicates a *p*-value < 0.001.

In the combined transcriptomic and metabolomic analysis, 8 KEGG pathways were found in the metabolome, and 21 in the transcriptome. Five of these KEGG pathways were shared by both the transcriptome and metabolome (Figure S4a). The O2PLS plot effectively reflects the consistency between the transcriptomic and metabolomic samples (Figure S4b). The combined transcriptomic and metabolomic analysis revealed highly significant enrichment of the Phenylpropanoid biosynthesis, Phenylpropanoid biosynthesis, and Linoleic acid metabolism KEGG pathways (*p* < 0.01). The Plant hormone signal transduction KEGG pathway was highly significantly enriched in the transcriptome samples (*p* < 0.01) and significantly enriched in the metabolome samples (*p* < 0.05). The Flavonoid biosynthesis KEGG pathway was highly significantly enriched in the transcriptome samples (*p* < 0.01) and showed a trend towards enrichment in the metabolome samples (*p* = 0.09) (Figure 7).

## 4. Discussion

### 4.1. Summer Heat Stress Poses a Serious Threat to Edible Grass

EG are perennial herbaceous plants, and their ability to endure continuous cutting is fundamental to their high biomass, which is the primary concern for this species under optimal cultivation conditions [5,7]. However, the yield data from this study clearly indicate that EG in the Hunan region face significant production reductions during the summer months.

Plants experience heat stress at both the canopy and leaf levels when environmental temperatures exceed their optimal physiological range. As summarized by Singsaas et al. [21], key drivers of this stress include elevated ambient air temperatures and, crucially, absorbed solar radiation, which can cause leaf temperature to rise significantly above air temperature [22]. The experimental site in the Hunan region is characterized by a subtropical monsoon climate with intense summer sunlight. Our empirical data confirm that under natural light (CK) conditions, the plants were subjected to such an environment: the maximum air temperature reached 38.85 °C, while the leaf temperature reached 38.14 °C. These temperatures are well above the optimal range for many temperate plants and are known to induce heat stress [9,10]. Therefore, the CK group in our study represents a field-realistic scenario of high light-aggravated heat stress, primarily driven by high light intensity, which leads to elevated leaf temperatures.

Exposure to heat stress triggers the overproduction of reactive oxygen species ROS, leading to oxidative stress that significantly damages cell membranes and protein conformations in plant leaf tissues [22]. The notable downregulation of heat shock protein (HSP) genes in the shading treatment group provides molecular evidence that the protein-folding environment was stabilized. While HSPs are essential for protecting proteins from heat-induced denaturation [23], their reduced expression under shading confirms that the treatment effectively alleviated the core protein homeostasis stress faced by plants in natural light condition. This hormonal interplay is reflected in our transcriptome data. The enrichment of the MAPK signaling pathway and the differential expression of key transcription factors such as ERF1, EIN3, and MYC2 indicate a significant reconfiguration of ethylene and jasmonic acid signaling in response to heat stress [24]. Our data directly link this yield decline to heat stress. We observed a significant inhibition of photosynthesis in the control group, a well-documented response to high temperatures [25], which was accompanied by clear signs of oxidative damage. This physiological disruption severely impaired biomass accumulation, identifying heat stress as the primary factor limiting yield. The efficacy of shading in alleviating this stress underscores its value as an agronomic strategy.

### 4.2. Alleviation of High Light–Aggravated Heat Damage in Edible Grass by Shading

The light saturation point for most vegetable crops ranges from 30,000 to 60,000 lux. For instance, the light saturation point is 30,000 lux for chili peppers, 60,000 lux for tomatoes, and 40,000 lux for eggplants. However, in Hunan Province, the maximum light intensity can exceed 100,000 lux on sunny summer days, [26]. Lower intensity light treatments may enable plants to produce photosynthetic products and accumulate biomass over an extended period. Conversely, when light intensity surpasses a certain threshold, photoinhibition occurs, leading to reduced photosynthesis, dehydration, wilting of leaves, and fruit scorching, which adversely affects the normal growth and development of vegetables [27]. Furthermore, according to [28], under high-intensity light conditions, the photochemical efficiency of plant leaves may degrade, inducing oxidative stress responses that negatively impact adversely affecting the physiological functions of plants. Concurrently, heat stress effects induced by sustained high-temperature environments can further disrupt plant physiological functions. Additionally, high temperatures combined with strong light can increase cellulose content, resulting in reduced CO_2_ assimilation rates and root vitality, while transpiration rates increase, leading to a decrease in the water content of vegetables.

This viewpoint is supported by related research. For instance, the study by Sage et al. [29] found that under conditions of high temperature and intense light, photosynthesis in plants is suppressed, leading to a decline in CO_2_ assimilation rates and an increase in and plant respiration, which results in higher energy consumption. Additionally, in environments characterized by elevated temperatures and strong light, the vitality of plant roots significantly decreases, while transpiration rates markedly increase, primarily due to damage to the membrane structures of plant cells caused by. these conditions, which adversely affect the absorption function of the roots [30]. These findings indicate that high temperatures and intense light can negatively impact plant growth and development in multiple ways. For instance, heat stress has been shown to reduce chlorophyll content in multiple studies. Heat stress-induced chloroplast damage downregulates important chloroplast components and inactivates heat-sensitive proteins such as RuBisCo activators, leading to redox imbalance, reduced photosynthetic efficiency, and potentially even cell death [31]. Furthermore, high-temperature stress can also reduce chlorophyll content by damaging the thylakoid membranes [32].

Utilizing shade nets for shading treatment is a cost-effective and efficient strategy to mitigate the high light-aggravated heat stress experienced by plants in summer and optimize the planting patterns [33]. Moderate shading has been shown to enhance the photosynthetic performance of vegetables, with the chlorophyll content increasing within a certain range of shading [32]. Research indicates 40–50% shading conditions, total chlorophyll content, chlorophyll a, chlorophyll b, and stomatal conductance all exhibit an upward trend. Furthermore, a black shade net with a light blocking rate of either 25% or 75% does not significantly enhance crop yield; however, a 50% shading rate can create a favorable microclimate [34,35]. Han et al. [36] found that shading increased the actual net photosynthetic rate of leaves by 16.8%, significantly enhancing chlorophyll content while reducing the accumulation of malondialdehyde (MDA) by 22%. In summary, summer shading treatment can improve plant photosynthesis [35], increase crop yield [34,37], and enhance quality [38,39].

This is consistent with our research findings, which indicate that shading treatment reduces both air and leaf temperature of forage grass, while significantly enhancing the stomatal conductance and intercellular CO_2_ concentration in the leaves. Consequently, this leads to an increased photosynthetic rate of the forage grass. This study suggests that the primary mechanism behind this phenomenon is the reduction in the photosynthetic rate under strong light conditions due to light inhibition. Following shading treatment, the reduced light intensity improves light energy utilization efficiency. Furthermore, shading decreases both light intensity and temperature, which in turn reduces leaf transpiration. When water supply is adequate, the stomata of the plants can remain open, facilitating the smooth entry of carbon dioxide into the leaf interior for photosynthesis, thereby enhancing the net photosynthetic rate.

### 4.3. Integrated Transcriptomic and Metabolomic Analyses Reveal a Shift from Defense to Growth Under Shading

In China, the EG has emerged as a novel resource for food ingredients, attracting significant interest in both food and animal feed sectors. However, the absence of a publicly available reference genome at the chromosomal level for EG has hindered comprehensive research into its molecular breeding and cultivation techniques. This study employed the Illumina HiSeq platform to perform paired-end RNA sequencing (PE150) on the leaves of EG cultivated under natural light and shading treatments in a subtropical monsoon climate zone, with the aim of elucidating its gene expression patterns under heat stress.

Initially, quality control of the original data was conducted, followed by de novo transcriptome assembly after the removal of low-quality reads. Subsequently, low-abundance transcripts (TPM < 1) and redundant sequences (with a threshold of ≥ 95% identity) were eliminated, resulting in a final assembly comprising 86,785 unigenes, with an N50 of 1546 bp. Phylogenetic BLAST similarity analysis indicated that the transcriptome of the EG exhibits high homology with sweet beet (*Beta vulgaris*) [40], quinoa (*Chenopodium quinoa*) [41], spinach (*Spinacia oleracea*) [42], and giant cactus (*Carnegiea gigantea*) [43] of the Cactaceae family, suggesting a relatively close evolutionary relationship. Furthermore, the EG belongs to the genus *Rumex* within the family Polygonaceae, providing a suitable close-relative reference for its gene functional annotation.

Our multi-omics approach provides a systems-level understanding of how shading reprograms the molecular landscape of EG, steering it away from a costly defense mode and towards a resource-efficient growth mode. The transcriptomic and metabolomic data are highly concordant, painting a coherent picture of this physiological shift.

The response of plants to heat stress is a complex phenomenon, and various techniques can be employed to analyze the underlying principles from multiple perspectives. RNA sequencing (RNA-Seq) has emerged as an effective method for exploring the mechanisms of plant abiotic stress and is widely utilized in horticulture. Metabolites often represent the final products of intricate biochemical cascade reactions associated with genomic, transcriptomic, and proteomic phenotypes [44]. Notably, the downregulation of flavonoid biosynthesis pathways in ST compared to CK, both at the gene expression level and in actual flavonoid metabolites, provides compelling evidence for the alleviation of high light-aggravated oxidative stress. Our transcriptomic data indicated significant enrichment that the KEGG pathway of “Flavonoid biosynthesis”. Importantly, our metabolomic analysis directly confirmed this at the metabolite level, revealing a significant reduction in the contents of flavonoids and related phenylpropanoids. Flavonoids are essential secondary metabolites synthesized in response to various abiotic stresses, including high light and heat, primarily functioning as antioxidants to scavenge reactive oxygen species (ROS) [45]. When faced with heat stress, reactive oxygen species (ROS) can rapidly surge, from various sources, primarily due to the over-reduction in the photosynthetic electron transport chain and the mitochondrial respiratory chain [46]. Additionally, wall peroxidases and NADPH oxidases located in the cytoplasm, plasma membrane, intercellular spaces, endoplasmic reticulum, and extracellular matrix of plant cells can also produce ROS [47]. The representative molecule H2O2 acts as a signaling substance that can initiate MAPK cascade reactions, cytoplasmic calcium flux, and the expression of heat shock transcription factors (such as HSFA1, HSFA2, etc.), thereby activating the plant’s defense system in response to external stress [48]. This process is accompanied by the elevated expression of antioxidant enzymes, including catalase (CAT), superoxide dismutase (SOD), ascorbate peroxidase (APX), and glutathione peroxidase (GPX), which eliminate excess H_2_O_2_ and •O_2_^−^. This action prevents oxidative stress-induced programmed cell death. When plants are subjected to heat stress, they activate a signaling network that spans organelles and pathways to trigger ROS bursts and initiate stress resistance responses. The main processes involved are ROS production, signal transduction, transcriptional responses, and defense execution. Our metabolomic analysis further supports this, revealing an altered profile in lipid metabolism-related metabolites, which may potentially contribute to enhanced membrane stability under shading.

Through the shading treatment of EG, we performed an advanced analysis of KEGG metabolic pathway enrichment, revealing that the expression of DEGs related to proteins or transcription factors is significantly downregulated throughout the entire heat stress response triggered by high light exposure (Figure 4a,b). Our results indicate that the ERF1 protein-related DEGs in the MAPK signaling pathway are significantly downregulated. Interestingly, the DEGs associated with the XRN4, EIN3, and MYC2 proteins are significantly upregulated. Generally, under high light or high temperature stress, the protein levels and activities of EIN3 and ERF1 are typically elevated, collectively initiating ethylene-mediated defense and adaptive responses. However, there are temporal differences and fine-tuned regulatory mechanisms of synergy and antagonism between the two under different stresses and in different tissues. Therefore, under heat stress, the ethylene signaling in the EG is enhanced: the C-terminal of EIN2 enters the nucleus to inhibit EBF1/2 and block the ubiquitin-mediated degradation of the EIN3 protein. This leads to the rapid accumulation of EIN3, which binds directly to the promoter of ERF1, initiating ERF1 transcription and activating defense genes such as PDF1.2 and PR1. This process enhances the plant’s tolerance to salt, heat, and strong light. Under shading, the activity of XRN4 increases significantly, accelerating the degradation of EBF1/2 mRNA and allowing EIN3 protein levels to recover. Meanwhile, the jasmonic acid signaling pathway is activated, causing MYC2 to competitively bind to the ERF1 promoter and inhibit its expression.

This results in a cross-regulation between JA and ET. Consequently, following shading treatment, a decoupling phenomenon is observed where EIN3 protein accumulates significantly while ERF1 transcription is suppressed. This case clearly demonstrates that when coping with field composite stress, internal hormone signaling pathways such as ethylene and jasmonic acid in plants do not operate independently but form a regulatory network with intricate synergistic and antagonistic relationships. This complex signal integration capability is key to plant adaptation to variable environments. Research indicates that similar signal crosstalk is widespread in different stress responses. For example, the core abscisic acid (ABA) signaling pathway also closely interacts with ethylene and jasmonic acid pathways during drought responses, collectively programming the plant’s overall stress resistance [49]. In summary, the differential upregulation of XRN4 confirms that the process of ‘inhibiting EIN3 degradation’ is actively amplified under shading conditions. This works in concert with MYC2 to precisely suppress ERF1, ultimately alleviating yield loss and mortality caused by heat stress.

Meanwhile, shading treatment resulted in a significant reduction in the expression of differentially expressed genes (DEGs) related to KatE (CAT1) and OXR1. Following the shading treatment, the expression of DEGs within the KEGG pathway associated with glutathione metabolism was notably downregulated. This included L-ascorbate peroxidase (APX), isocitrate dehydrogenase (IDH), 6-phosphogluconate dehydrogenase (6PGD), glutathione peroxidase (GPX), and glutathione-S-transferase-like or Prx family. Glutathione metabolism plays a vital role in the elimination of reactive oxygen species (ROS) and alleviates oxidative damage. Research indicates that glutathione (GSH) is one of the most important intracellular antioxidants, capable of scavenging ROS through the GSH-GSSG cycle. This process reduces or prevents oxidative stress and cellular damage [50]. Furthermore, glutathione is an essential component of the plant antioxidant system, playing critical roles in detoxifying reactive oxygen species and methylglyoxal under stress conditions [51]. For example, in plants, glutathione metabolism is integral to maintaining intracellular redox balance, particularly in response to environmental stress, by regulating the activity of antioxidant enzymes to eliminate excess ROS [52]. A study on Agave americana in the United States, revealed that under osmotic stress, by modulating the activity of antioxidant enzymes such as superoxide dismutase (SOD), plants can enhance the content of reduced glutathione (GSH), thereby eliminating reactive oxygen species and reactive carbonyl species, reducing lipid peroxidation levels, and improving stress resistance [53].

The KEGG metabolic related to pathway of protein processing in the endoplasmic reticulum was found to be significantly enriched. Notably, we observed that the expression levels of DEGs such as Hsp40, Hsp70, and Hsp90 were downregulated under shading treatment. These proteins are well-known members of the Heat Shock Proteins (Hsps) family, which are highly conserved molecular chaperones that are induced under heat stress and other abiotic stresses. They play a crucial role in assisting with the correct folding, transport, repair, or degradation of proteins, thereby maintaining cellular protein homeostasis and enhancing plant stress resistance [10]. The downregulation of these protein DEGs suggests that shading reduced the leaf temperature of the forage, decreased photoinhibition, and minimized reactive ROS accumulation. This led the plants to not perceive intense heat or oxidative stress, indicating that there was no requirement for extensive Hsps for protein repair.

In summary, shading shifts the mode of the EG from “ROS stress-high energy defense” to “low oxidative pressure-resource conservation.” This transition reduces the necessity for substantial amounts of CAT1 and OXR1, as well as the high expression of the entire GSH antioxidant module. The conserved carbon and nitrogen/reducing power can be directly utilized for growth, thereby alleviating yield loss and mortality caused by lack of shading. Therefore, by integrating metabolic pathway data with relevant physiological data, we propose that, following shading treatment, EG responds to stress by regulating photosynthesis-antenna proteins and related metabolic pathways, such as the MAPK signaling pathway and glutathione metabolism, to enhance its stress resistance. Shading treatment not only optimizes energy allocation but also reduces oxidative damage by downregulating genes related to ROS production, thereby enhancing the survival ability of EG in stressful environments. This multi-layered regulatory mechanism provides new insights for research into plant stress resistance.

## 5. Conclusions

The subtropical monsoon climate region is characterized by high light levels, elevated temperatures and intense UV radiation during the summer months. These conditions lead to an immediate outbreak of reactive oxygen species (ROS) in herbaceous plants. prompting the activation of their defense systems to mitigate thermal stress. Consequently, this results in a notable reduction in both the yield and quality of the plants. Comparative transcriptomic analyses indicate that, under shading conditions, oxidative stress is associated with a notable downregulation of antioxidant genes, such as CAT1, OXR1, APX and GPX, as well as heat stress protein genes, including Hsp40, Hsp70, Hsp90, and UbcH5, and genes involved in flavonoid synthesis genes. This transcriptional reprogramming is further substantiated by metabolomic findings, which reveal a substantial decrease in the accumulation of flavonoid antioxidants. Such observations suggest that the ROS pressure has been alleviated and that high-energy defense mechanisms are unnecessary. Additionally, MYC2 is significantly upregulated, which inhibits ERF1 and mitigates excessive ethylene defense. Concurrently, XRN4 is upregulated to stabilize EIN3; however, its downstream target ERF1 is suppressed by MYC2, resulting in a ‘low defense–high growth’ state. Additionally, the observed upregulation of lipid metabolism-related metabolites provides further support for enhanced membrane stability under shading conditions. Ultimately, photosynthetic recovery occurs, leading to decreased ROS levels and eliminating the need for high expression of antioxidant and flavonoid enzymes. As a result, conserved carbon, nitrogen, and reducing power are reinvested in growth, achieving a reallocation of carbon flow and energy.

## Figures and Tables

**Table 1:** The Impact of Shading Treatment on the Yield and Quality of Edible Grass. CK represents the Control group, while ST denotes the shading treatment group.

Parameter	CK Group	ST Group	*p* Value
Plant height, cm	30.53 ± 4.92	54.08 ± 4.85	<0.001
Leaf weight, cm	7.68 ± 1.26	12.13 ± 2.24	<0.001
Fresh weight, t/hm ^2^	8.49 ± 0.81	16.31 ± 0.72	<0.001
Dry weight, t/hm ^2^	1.07 ± 0.11	1.64 ± 0.11	<0.001
Dry matter, g/kg	12.63 ± 0.47	10.08 ± 0.23	<0.001
Nitrogen content, g/kg	35.66 ± 1.85	43.42 ± 2.74	<0.001
Crude protein, g/kg	222.88 ± 11.55	271.35 ± 17.16	<0.001
Neutral detergent fiber, g/kg	443.14 ± 19.44	412.08 ± 15.36	<0.001
Acid detergent fiber, g/kg	288.57 ± 14.87	256.79 ± 12.58	<0.001

**Table 2:** The impact of shading treatment on the antioxidant characteristics of edible grass. CK represents the Control group, while ST denotes the shading treatment group.

Parameter	CK Group	ST Group	*p* Value
SOD activity, U/g FW	11,607.66 ± 197.96	10,863.76 ± 225.72	<0.001
APX activity, U/g FW	6.12 ± 0.77	3.95 ± 0.77	0.002
POD activity, U/g FW	600.26 ± 29.08	441.83 ± 14.25	<0.001
CAT activity, U/g FW	23.75 ± 1.84	17.90 ± 2.84	0.005
MDA, nmol/g FW	9.44 ± 1.72	4.36 ± 0.56	<0.001
H_2_O_2_, μmol/g FW	13.03 ± 1.71	7.40 ± 2.06	0.006
O_2_^−^ content, μmol/g FW	19.48 ± 0.65	12.85 ± 0.48	0.002

Notes: Superoxide Dismutase (SOD), Catalase (CAT), Ascorbate Peroxidase (APX) and Peroxidase (POD), as well as the Contents of Malondialdehyde (MDA), Hydrogen Peroxide (H_2_O_2_), and Superoxide Anion (·O_2_^−^).

**Table 3:** The impact of shading treatment on the photosynthetic parameters of edible grass. CK represents the Control group, while ST denotes the shading treatment group.

Parameter	CK Group	ST Group	*p* Value
Net photosynthetic rate, mmol/(m^2^·s)	8.93 ± 1.20	10.88 ± 1.86	<0.001
Stomatal conductance, mmol/(m^2^·s)	0.32 ± 0.06	0.58 ± 0.27	<0.001
Intercellular CO2 concentration, μmol/(m^2^·s)	357.93 ± 8.30	393.24 ± 17.90	<0.001
Rate of transpiration, mmol/(m^2^·s)	38.97 ± 0.48	35.76 ± 1.67	<0.001
Air temperature, ℃	38.85 ± 0.50	36.48 ± 0.51	0.857
Leaf temperature, ℃	38.14 ± 0.93	35.18 ± 0.76	0.005

**Table 4:** Presents the statistical data of transcript assembly.

Parameter	Unigene of Raw Assembly	Transcript of Raw Assembly	Unigene of Filtere Assembly	Transcript of Filtere Assembly
Total number	103,182	279,651	86,785	192,766
Total base	82,813,202	334,732,670	78,369,704	217,743,050
Largest length (bp)	21,819	21,819	21,819	21,819
Smallest length (bp)	201	201	201	201
Average length (bp)	802.59	1196.97	903.03	1129.57
N50 length (bp)	1428	1979	1546	1769
E90 length (bp)	2631	2128	2622	2076
Fragment mapped percent (%)	54.512	80.17	54.593	78.973
GC percent (%)	40.6	41.46	40.72	41.34
TransRate score	0.22187	0.325	0.29065	0.42604
Complete BUSCO (%)	70.9%	86.8%	71.4%	86.6%
Single-copy gene BUSCO (%)	67.5%	10.8%	68.1%	23.5%
Duplicate BUSCO (%)	3.4%	76.0%	3.3%	63.1%

## Data Availability

The original contributions presented in this study are included in the article/supplementary material. Further inquiries can be directed to the corresponding authors.

## Supplementary Materials

The following supporting information can be downloaded at: https://www.mdpi.com/article/10.3390/antiox15010033/s1, Table S1. RNA-seq sequencing output and quality control metrics. CK represents the Control group, while ST denotes the shading treatment group. Table S2. Summary of read mapping rates to the de novo assembled transcriptome. CK represents the Control group, while ST denotes the shading treatment group. Figure S1. Bar chart of annotation information from different databases. Figure S2. Functional classification of the edible grass transcriptome. Figure S3. Functional annotation of the EG de novo transcriptome assembly. Figure S4. Overview of combined transcriptomic and metabolomic analysis.

## References

[1] He T., Li X., Wang Z., Mao J., Mao Y., Sha R. (2024). Studies on the Changes of Fermentation Metabolites and the Protective Effect of Fermented Edible Grass on Stress Injury Induced by Acetaminophen in HepG2 Cells. Foods.

[2] Wang X., Wang J., Liu Y., Li G., Gong S., Wang H., He D. (2025). Exploring the Role of Edible Dock Powder (Rumex K-1) in Enhancing Growth Performance, Organ Health, and Cecal Microbiota in Sanhua Goslings. Agriculture.

[3] Li X., He T., Mao Y., Mao J., Lai X., Tu H., Zhou Y., Sha R. (2023). Changes in Physicochemical Properties, Metabolites and Antioxidant Activity of Edible Grass during Spontaneous Fermentation. Fermentation.

[4] Jiang T., Li X., Wang H., Pi M., Hu J., Zeng Z., Li B., Xu Z. (2024). Identification and quantification of flavonoids in edible dock based on UPLC-qTOF MS/MS and molecular networking. J. Food Compos. Anal..

[5] Ślesak H., Liszniańska M., Popielarska-Konieczna M., Góralski G., Sliwinska E., Joachimiak A.J. (2014). Micropropagation protocol for the hybrid sorrel *Rumex tianschanicus* × *Rumex patientia*, an energy plant. Histological, SEM and flow cytometric analyses. Ind. Crop. Prod..

[6] He Z., Zou J., Li X., Jiang T., Zeng J., Chen M. (2025). Responses of nitrogen metabolism, photosynthetic parameter and growth to nitrogen fertilization in *Rumex patientia* L. × *Rumex tianschanicus* Losinsk. Chil. J. Agric. Res..

[7] Mareri L., Parrotta L., Cai G. (2022). Environmental Stress and Plants. Int. J. Mol. Sci..

[8] Zhao J., Lu Z., Wang L., Jin B. (2021). Plant Responses to Heat Stress: Physiology, Transcription, Noncoding RNAs, and Epigenetics. Int. J. Mol. Sci..

[9] Hasanuzzaman M., Nahar K., Alam M.M., Roychowdhury R., Fujita M. (2013). Physiological, Biochemical, and Molecular Mechanisms of Heat Stress Tolerance in Plants. Int. J. Mol. Sci..

[10] Xalxo R., Yadu B., Chandra J., Chandrakar V., Keshankant S., Wani S.H., Kumar V. (2020). Alteration in Carbohydrate Metabolism Modulates Thermotolerance of Plant under Heat Stress. Heat Stress Tolerance in Plants: Physiological, Molecular and Genetic Perspectives.

[11] Liu J., Zhang R., Xu X., Fowler J., Miller T., Dong T. (2020). Effect of summer warming on growth, photosynthesis and water status in female and male *Populus cathayana*: Implications for sex-specific drought and heat tolerances. Tree Physiol..

[12] Jespersen D., Hossain M.A., Liu F., David J., Burritt D. (2020). Heat shock induced stress tolerance in plants: Physiological, biochemical, and molecular mechanisms of acquired tolerance. Priming-Mediated Stress and Cross-Stress Tolerance in Crop Plants.

[13] Siddique A.B., Shabala S., Li C., Chen Z., Varshney R., Zhao C., Zhou M. (2025). Reducing heat stress damage in cereal crops through agronomic management and breeding strategies. Plant Stress.

[14] Pallotti L., Silvestroni O., Dottori E., Tania L., Lanari L. (2023). Effects of shading nets as a form of adaptation to climate change on grapes production: A review. Oeno One.

[15] Milenkovic L., Ilic Z., Durovka M., Kapoulas N., Mirecki N., Fallik E. (2012). Yield and pepper quality as affected by light intensity using colour shade nets. Agric. Forest.

[16] AOAC (1995). Official Methods of Analysis of AOAC International.

[17] Van Soest P.J., Robertson J., Lewis B. (1991). Methods for dietary fiber, neutral detergent fiber, and nonstarch polysaccharides in relation to animal nutrition. J. Dairy. Sci..

[18] Tao L., Long Q., Shang Q., Zhang Q., Guo G., Cai H., Geng J., Song X., Zeng H., Wang W. (2025). Comprehensive Transcriptome and Metabolome Analysis Reveals the Potential Mechanism Influencing Flower Color Formation in *Macadamia integrifolia*. Horticulturae.

[19] Love M., Huber W., Anders S. (2014). Moderated estimation of fold change and dispersion for RNA-seq data with DESeq2. Genome biol..

[20] Hinderer I., Moseley H. (2020). GOcats: A tool for categorizing Gene Ontology into subgraphs of user-defined concepts. PLoS ONE.

[21] Singsaas E., Laporte M., Jain S., Monson R., Bowling D., Kristine J., Manuel L., Amal J., Sharkey T. (1991). Kinetics of leaf temperature fluctuation affect isoprene emission from red oak (*Quercus rubra*) leaves. Tree Physiol..

[22] Liu M., Liu X., Song Y., Hu Y., Yang C., Li J., Jin S., Gu K., Yang Z., Huang W. (2024). Tobacco production under global climate change: Combined effects of heat and drought stress and coping strategies. Front. Plant Sci..

[23] Ahanger M., Akram N., Ashraf M., Alyemeni M., Wijaya L., Ahmad P. (2017). Plant responses to environmental stresses—From gene to biotechnology. AoB Plants.

[24] Li N., Euring D., Cha J., Lin Z., Lu M., Huang L., Kim W. (2021). Plant hormone-mediated regulation of heat tolerance in response to global climate change. Front. Plant Sci..

[25] Djanaguiraman M., Narayanan S., Erdayani E., Prasad P. (2020). Effects of high temperature stress during anthesis and grain filling periods on photosynthesis, lipids and grain yield in wheat. BMC Plant Biol..

[26] Rubatzky V., Yamaguchi M. (2012). World Vegetables: Principles, Production, and Nutritive Values.

[27] Eghbal E., Aliniaeifard S., Mehrjerdi M., Abdi S., Hassani S., Rassaie T., Gruda N. (2024). Growth, phytochemical, and phytohormonal responses of basil to different light durations and intensities under constant daily light integral. BMC Plant Biol..

[28] Yang B., Tang J., Yu Z., Khare T., Srivastav A., Datir S., Kumar V. (2019). Light stress responses and prospects for engineering light stress tolerance in crop plants. J. Plant Growth Regul..

[29] Sage R.F., Kubien D.S. (2007). The temperature response of C3 and C4 photosynthesis. Plant Cell Environ..

[30] Liu X., Huang B. (2000). Heat stress injury in relation to membrane lipid peroxidation in creeping bentgrass. Crop Sci..

[31] Zahra N., Hafeez M.B., Ghaffar A., Kausar A., Al Zeidi M., Siddique K.H., Farooq M. (2023). Plant photosynthesis under heat stress: Effects and management. Environ. Exp. Bot..

[32] Sachdev S., Ansari S.A., Ansari M.I., Fujita M., Hasanuzzaman M. (2021). Abiotic Stress and Reactive Oxygen Species: Generation, Signaling, and Defense Mechanisms. Antioxidants.

[33] Xie A., Lv M., Zhang D., Shi Y., Yang L., Yang X., Du J., Sun L., Sun X. (2023). Effects of slight shading in summer on the leaf senescence and endogenous hormone and polyamine contents in herbaceous peony. Sci. Rep..

[34] Zeng G., Guo Y., Xu J., Hu M., Zheng J., Wu Z. (2017). Partial shade optimizes photosynthesis and growth in bayberry (*Myrica rubra*) trees. Hortic. Environ. Biotechnol..

[35] Ilic Z., Fallik E. (2017). Light quality manipulation improves vegetable quality at harvest and postharvest: A review. Environ. Exp. Bot..

[36] Han C., Wang Q., Zhang H., Wan S., Song H., Hao J., Dong H. (2018). Light shading improves the yield and quality of seed in oil-seed peony (*Paeonia ostii* Feng Dan). J. Integr. Agric..

[37] Oliveira G., Vieira W., Bertolli S., Pacheco A. (2016). Photosynthetic behavior, growth and essential oil production of Melissa officinalis L. cultivated under colored shade nets. Chil. J. Agric. Res..

[38] AIlic Z., Milenkovic L., Sunic L., Fallik E. (2015). Effect of coloured shade-nets on plant leaf parameters and tomato fruit quality. J. Sci. Food Agr..

[39] Buthelezi M., Soundy P., Jifon J., Sivakumar D., Millicent N., Soundy P., Jifon J., Sivakumar D. (2016). Spectral quality of photo-selective nets improves phytochemicals and aroma volatiles in coriander leaves (*Coriandrum sativum* L.) after postharvest storage. J. Photochem. Photobiol..

[40] McGrath J., Funk A., Galewski P., Ou S., Townsend B., Davenport K., Daligault H., Johnson S., Lee J., Hastie A. (2023). A contiguous de novo genome assembly of sugar beet EL10 (*Beta vulgaris* L.). DNA Res..

[41] Bodrug-Schepers A., Stralis-Pavese N., Buerstmayr H., Dohm J., Himmelbauer H. (2021). Quinoa genome assembly employing genomic variation for guided scaffolding. Theor. Appl. Genet..

[42] Zhao Y., Liu Z., She H., Xu Z., Zhang H., Zheng S., Qian W. (2025). Comparative Transcriptome Analysis of Gene Expression Between Female and Monoecious *Spinacia oleracea* L. Genes.

[43] Yan J., Yu L., Xuan J., Lu Y., Lu S., Zhu W. (2016). De novo transcriptome sequencing and gene expression profiling of spinach (*Spinacia oleracea* L.) leaves under heat stress. Sci. Rep..

[44] Nover L., Scharf K., Roberto B., Jean L. (2024). Heat stress proteins and transcription factors. Cellular and Molecular Life Sciences.

[45] Shomali A., Das S., Arif N., Sarraf M., Zahra N., Yadav V., Aliniaeifard S., Chauhan D.K., Hasanuzzaman M. (2022). Diverse Physiological Roles of Flavonoids in Plant Environmental Stress Responses and Tolerance. Plants.

[46] Fortunato S., Lasorella C., Dipierro N., Vita F., de Pinto M.C. (2023). Redox Signaling in Plant Heat Stress Response. Antioxidants.

[47] Panda S.K., Gupta D., Patel M., Vyver C.V.D., Koyama H. (2024). Functionality of Reactive Oxygen Species (ROS) in Plants: Toxicity and Control in Poaceae Crops Exposed to Abiotic Stress. Plants.

[48] Batool I., Ayyaz A., Qin T., Wu X., Chen W., Hannan F., Zafar Z.U., Naeem M.S., Farooq M.A., Zhou W. (2025). Morphological, Physiological, and Molecular Responses to Heat Stress in Brassicaceae. Plants.

[49] Ma Y., Tang M., Wang M., Yu Y., Ruan B. (2024). Advances in Understanding Drought Stress Responses in Rice: Molecular Mechanisms of ABA Signaling and Breeding Prospects. Genes.

[50] Dorion S., Ouellet J.C., Rivoal J. (2021). Glutathione Metabolism in Plants under Stress: Beyond Reactive Oxygen Species Detoxification. Metabolites.

[51] Cotgreave I., Gerdes R. (1998). Recent trends in glutathione biochemistry—Glutathione-protein interactions: A molecular link between oxidative stress and cell proliferation?. Biochem. Biophys. Res. Commun..

[52] Kovacs I., Holzmeister C., Wirtz M., Geerlof A., Frohlich T., Roemling G., Kuruthukulangarakoola G., Linster E., Hell R., Arnold G. (2016). ROS-Mediated Inhibition of S-nitrosoglutathione Reductase Contributes to the Activation of Anti-oxidative Mechanisms. Front. Plant Sci..

[53] Shen Y., Di J., Chen Y., Feng K., Lu M., Hu Y. (2024). Effects of H_2_S donor NaHS on the adaptability and antioxidant properties of Agave americana plantlets under an in vitro culture of osmotic stress. J. Nanjing For. Univ..

