# Supplementary material for: Sustaining Edible Grass (Rumex patientia L. × Rumex tianschanicus Losinsk.) Through Summer Lethal Stress: Multi-Omics Reveals Shading-Mediated Mitigation of High Light-Aggravated Heat Damage
Source: Antioxidants (Basel). 2025 Dec 25;15(1):33. doi: 10.3390/antiox15010033 (PMC12837377; doi:10.3390/antiox15010033)
Supplement: Supplementary file 1 [file antioxidants-15-00033-s001.zip › antioxidants-3876529-supplementary.pdf]

Supplementary Table 1. RNA-seq sequencing output and quality control metrics. CK represents the Control group, while ST denotes the shading treatment group.

Group name	Sample name	No. of raw reads	No. of clean reads	% reads retained after filtering
CK Group	CK_1	44,762,606	44,444,556	99.29
	CK_2	44,226,024	43,925,836	99.32
	CK_3	43,311,342	43,002,018	99.29
	CK_4	43,265,356	42,965,150	99.31
	CK_5	40,879,094	40,580,306	99.27
	CK_6	39,931,698	39,648,280	99.29
ST Group	ZY_1	41,629,394	41,342,190	99.31
	ZY_2	40,954,812	40,657,674	99.27
	ZY_3	44,789,252	44,470,606	99.29
	ZY_4	39,461,786	39,190,358	99.31
	ZY_5	43,580,964	43,267,980	99.28
	ZY_6	43,853,708	43,550,102	99.31
Total		510,646,036	507,045,056	-

Supplementary Table 2. Summary of read mapping rates to the de novo assembled transcriptome.CK represents the Control group, while ST denotes the shading treatment group.

Group name	Sample name	No. of clean reads	Reads mapped (%)
CK Group	CK_1	22,222,278	85.92%
	CK_2	21,962,918	85.59%
	CK_3	21,501,009	85.13%
	CK_4	21,482,575	85.75%
	CK_5	20,290,153	85.18%
	CK_6	19,824,140	85.33%
ST Group	ST_1	20,671,095	84.54%
	ST_2	20,328,837	84.09%
	ST_3	22,235,303	84.29%
	ST_4	19,595,179	84.62%
	ST_5	21,633,990	84.67%
	ST_6	21,775,051	84.36%

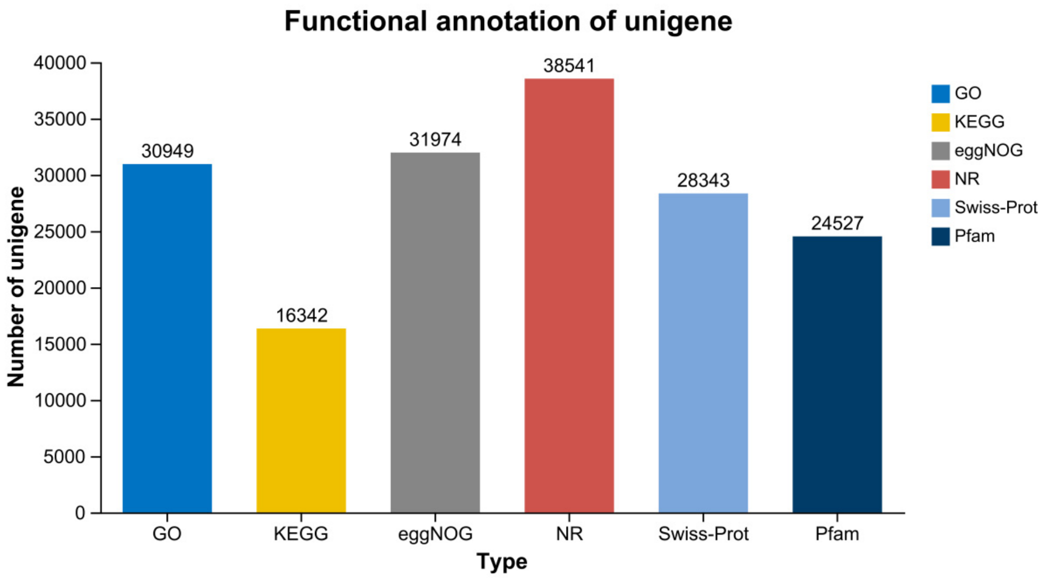

Supplementary Figure 1. Bar chart of annotation information from different databases.

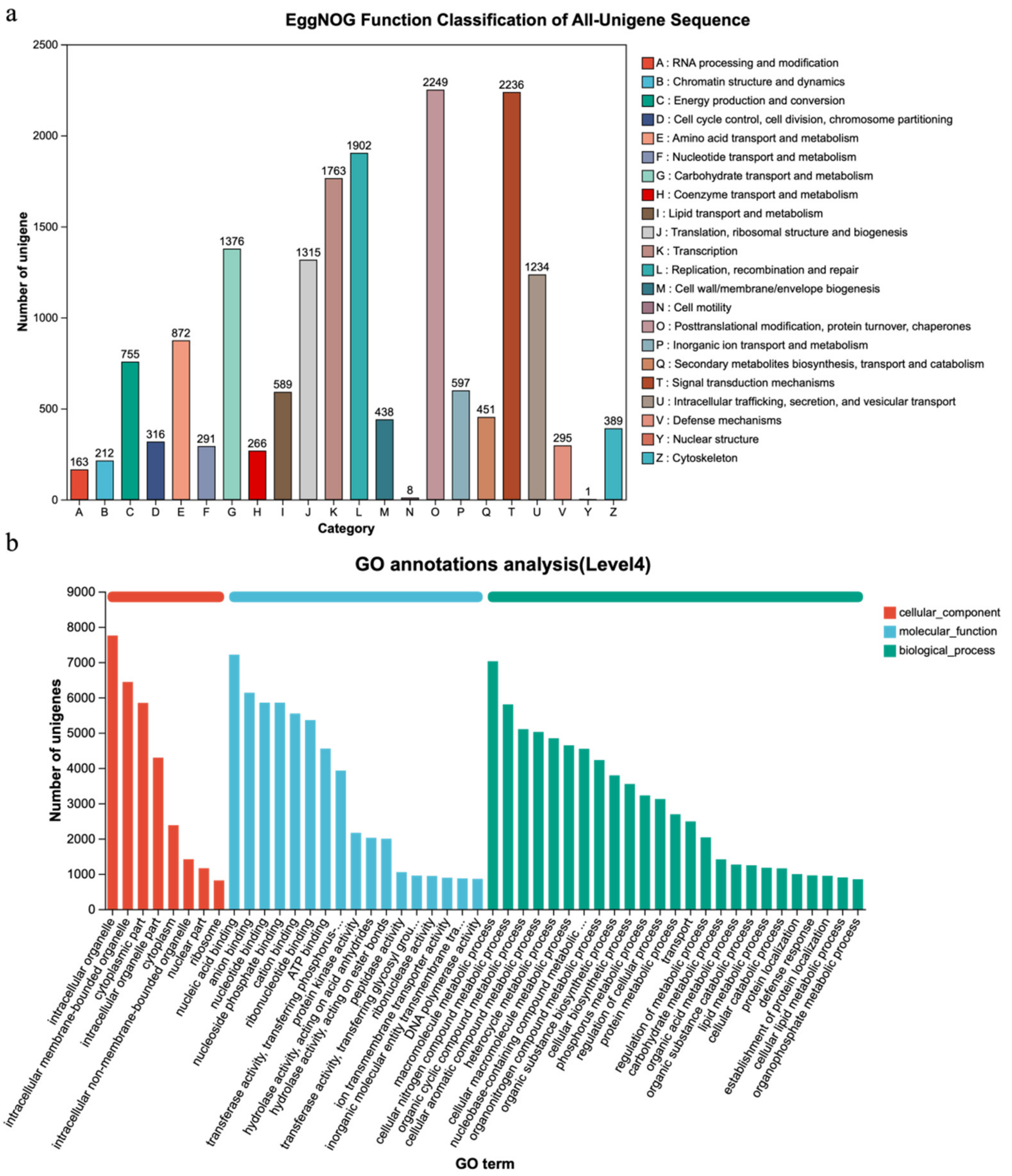

Supplementary Figure 2. Functional classification of the edible grass transcriptome. (a) Clustering of orthologous groups (COG) classification based on the eggNOG database. The histogram shows the distribution of 31,974 unigenes across 24 major functional categories. The top categories, excluding 'Function unknown', are labeled. (b) Gene Ontology (GO) classification of the assembled unigenes. A total of 30,949 unigenes were annotated and categorized into the three main GO ontologies: Biological Process, Molecular Function, and Cellular Component. The bar chart shows the number of unigenes associated with the most abundant terms within each ontology.

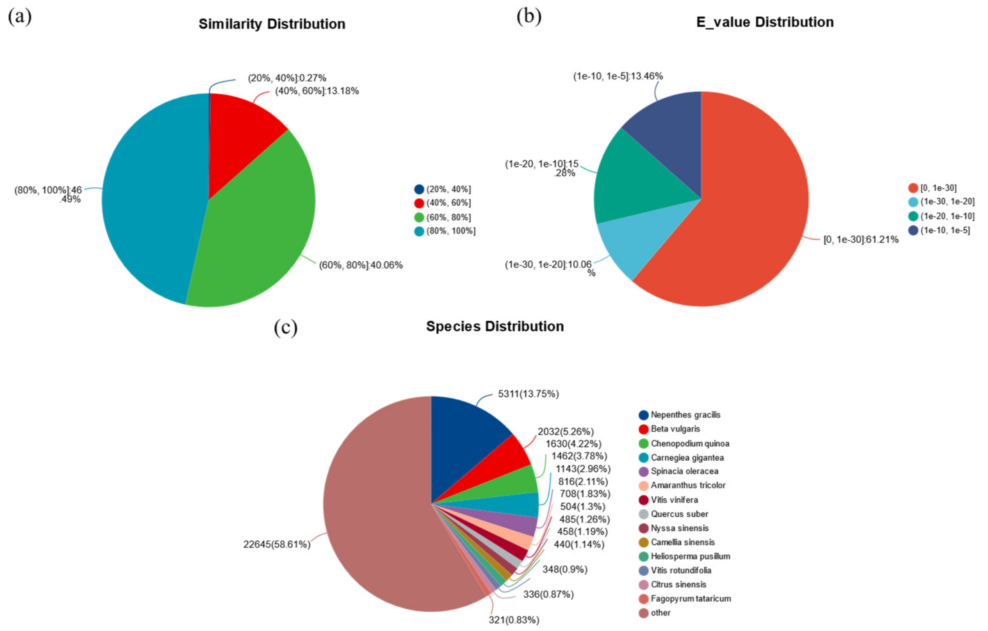

Supplementary Figure 3. Functional annotation of the EG *de novo* transcriptome assembly. (a) Distribution of E-values for BLASTX matches against the NCBI non-redundant (NR) protein database. The majority (61.21%) of annotated unigenes had highly significant matches (E-value < $1e^{-30}$). (b) Distribution of sequence similarity for the top BLAST hits in the NR database. Most unigenes (86.55%) showed sequence similarity greater than 60%. (c) Species distribution of the top BLAST hits, showing the proportion of unigenes with the highest sequence homology to five representative plant species.

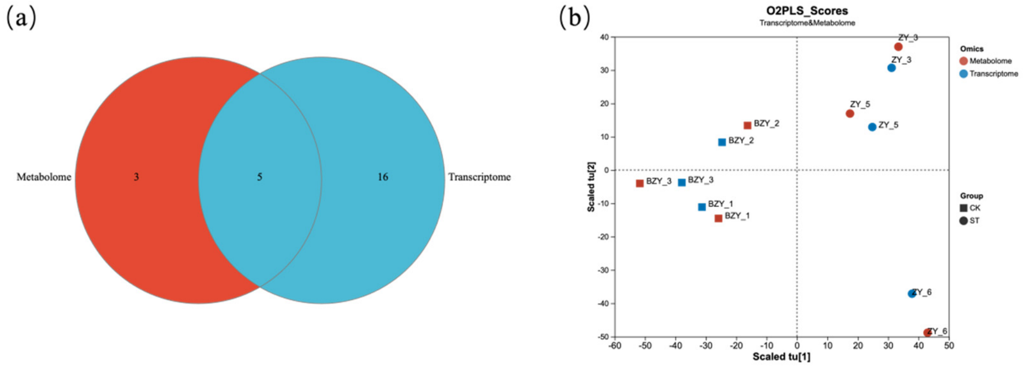

Supplementary Figure 4. Overview of combined transcriptomic and metabolomic analysis. (a) Venn plot of KEGG enrichment in transcriptomic and metabolomic analyses; (b) O2PLS plot of transcriptomic and metabolomic analyses. BZY-1, BZY-2, and BZY-3 represent samples from the CK group, and ZY-1, ZY-2, and ZY-3 represent samples from the ST group.
